# Small Molecule Inhibitors of the *Candida albicans* Budded-to-Hyphal Transition Act through Multiple Signaling Pathways

**DOI:** 10.1371/journal.pone.0025395

**Published:** 2011-09-26

**Authors:** John Midkiff, Nathan Borochoff-Porte, Dylan White, Douglas I. Johnson

**Affiliations:** Department of Microbiology and Molecular Genetics, University of Vermont, Burlington, Vermont, United States of America; University of Minnesota, United States of America

## Abstract

The ability of the pathogenic yeast *Candida albicans* to interconvert between budded and hyphal growth states, herein termed the budded-to-hyphal transition (BHT), is important for *C. albicans* development and virulence. The BHT is under the control of multiple cell signaling pathways that respond to external stimuli, including nutrient availability, high temperature, and pH. Previous studies identified 21 small molecules that could inhibit the *C. albicans* BHT in response to carbon limitation in Spider media. However, the studies herein show that the BHT inhibitors had varying efficacies in other hyphal-inducing media, reflecting their varying abilities to block signaling pathways associated with the different media. Chemical epistasis analyses suggest that most, but not all, of the BHT inhibitors were acting through either the Efg1 or Cph1 signaling pathways. Notably, the BHT inhibitor clozapine, a FDA-approved drug used to treat atypical schizophrenia by inhibiting G-protein-coupled dopamine receptors in the brain, and several of its functional analogs were shown to act at the level of the Gpr1 G-protein-coupled receptor. These studies are the first step in determining the target and mechanism of action of these BHT inhibitors, which may have therapeutic anti-fungal utility in the future.

## Introduction

*Candida albicans* is the most common causative agent of systemic human fungal infections [1]. It is a major opportunistic pathogen of immunosuppressed hosts, including AIDS patients and those undergoing chemotherapy or tissue transplants. In addition, *C. albicans* is the fourth leading cause of nosocomial bloodstream infections, especially in patients with indwelling medical devices [2]. Therefore, insights into the mechanisms by which *C. albicans* causes disease are likely to lead to the development of new prophylactic and therapeutic strategies.

*C. albicans* cells exist in different morphological and developmental states, including a budded (yeast-like) form and both pseudohyphal and true hyphal filamentous forms. The ability to switch between budded and hyphal morphological states, referred herein as the budded-to-hyphal transition (BHT), occurs in response to a variety of external signals including elevated temperature or pH, nitrogen and/or carbon starvation, and the presence of host macrophages [3]–[5]. The most potent inducer of the BHT is growth in complex media containing 10% serum at 37°C, however the precise component of serum responsible for the induction is unknown. Growth in other nutrients such as *N*-acetylglucosamine (GlcNAc), methionine, or proline, or in nutrient-poor media such as Lee's, Spider, and low-nitrogen SLAD media [6], [7] can also induce the BHT. The BHT is also affected in response to alkaline pH, osmotic stress and reactive oxygen species (ROS), suggesting that BHT induction mimics a stress response in many regards.

**Figure 1:**
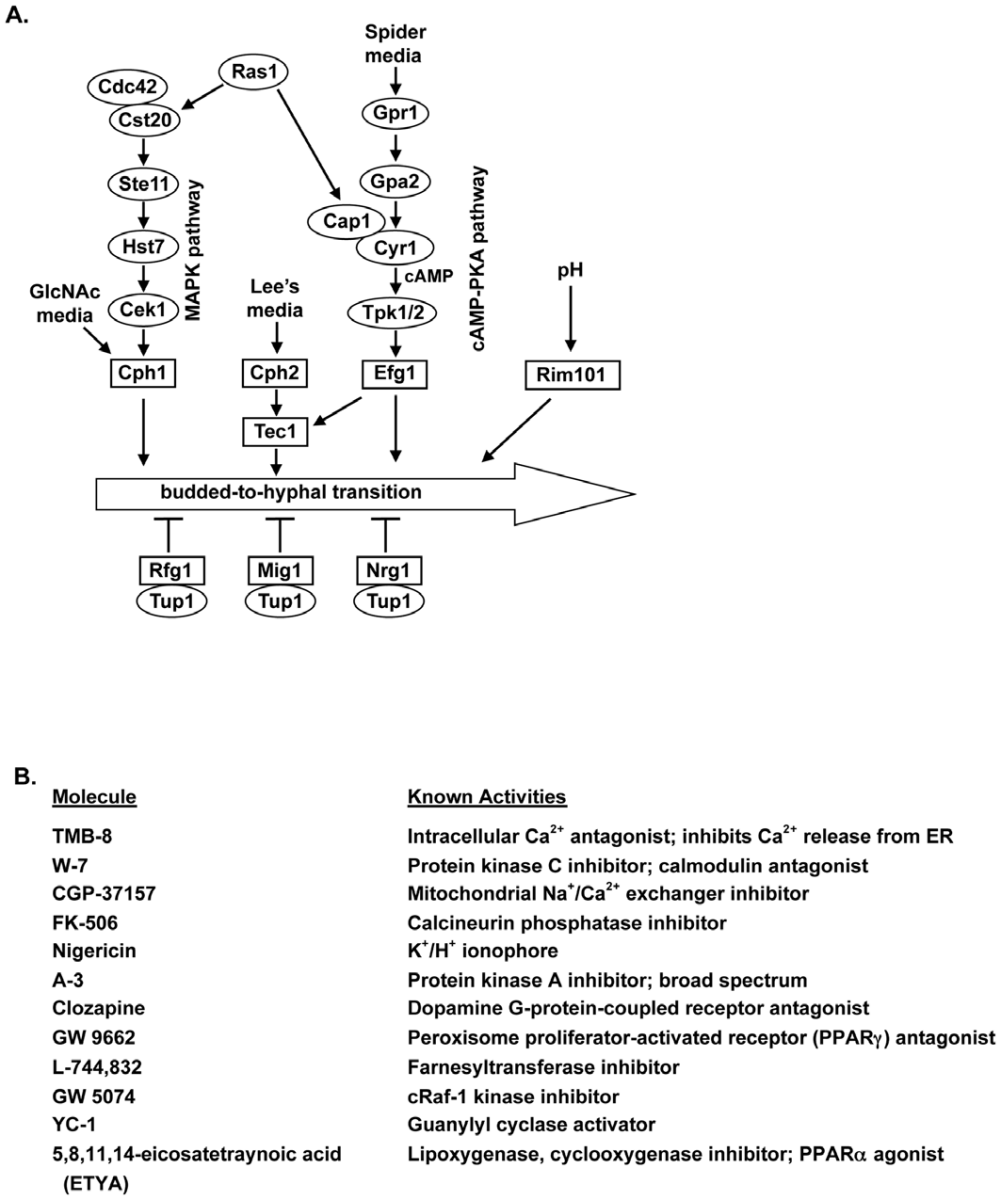
BHT pathways and inhibitors. **A,** Morphological signaling pathways in *C. albicans*. For simplicity, only the Efg1, Cph1, Cph2/Tec1, and Rim101 pathways are shown. **B,** BHT inhibitors used in this study.

Multiple intracellular signaling pathways respond to these different growth and environmental signals to induce or repress hyphal development [8]–[10] (Fig. 1A). Each of these pathways activates different transcription factors (Fig. 1A, square boxes) that induce expression of hyphal-specific genes. *C. albicans* cells respond to temperature, serum, and glucose through the Ras1 GTPase [11], [12]. Activated GTP-bound Ras1 interfaces with two signaling pathways: the Cek1 MAP kinase pathway and the protein kinase A (PKA) pathway (Fig. 1A). The Cek1 MAPK pathway signals to the Cph1 transcription factor [7], [13]–[15], whereas the cAMP-PKA pathway signals to the Efg1 transcription factor. Ras1 activates adenylyl cyclase (Cyr1), generating a cAMP signal that leads to the activation of the Tpk1 and Tpk2 catalytic subunits of PKA [16], which in turn phosphorylate and activate the Efg1 transcription factor [17], [18]. The Efg1 pathway is also responsive to certain amino acids, such as methionine, and carbon deprivation (*i.e.*, growth on Spider media) through the Gpr1 G-protein-coupled receptor and its G-alpha subunit Gpa2 [19]–[22].

The BHT is also induced by GlcNAc through the GlcNAc transporter Ngt1 [23] and an ill-defined signaling pathway that activates the Cph1 transcription factor [24], [25]. The Cph2 and Tec1 transcription factors respond to a variety of growth media including the amino acids in Lee's media [26]–[29]. Cells respond to changes in pH through the Rim101 transcription factor [30], [31]. While these pathways can be activated by separate but overlapping developmental signals, the cross-talk between the pathways has not been fully elucidated. The Tup1 BHT repressor is constitutively expressed but it functions with three other negative regulators, Nrg1, Mig1, and Rfg1 to repress the BHT [32]–[34]. However, for the purposes of this study, we focused on signaling pathways that induce (not repress) the BHT, as the inhibitors we have identified most likely function through blocking one or more of these pathways.

Previously, we identified five novel small molecules and 16 molecules with known mammalian targets or mechanisms of action that could inhibit the BHT without affecting budded growth [35], [36]. The known molecules affect various signaling pathway components in mammalian cells, including those involved in calcium homeostasis, cAMP-dependent protein kinase (PKA) and other kinases, G-protein-coupled receptors and other receptors, Ras GTPase signaling, and other signaling proteins [36]. This is particularly encouraging as the roles of PKA, the G-protein-coupled receptor Gpr1, and the Ras1 GTPase in the Efg1 pathway are well established [11], [16], [19] (Fig. 1A). The BHT inhibitors were identified in assays utilizing carbon-limiting Spider media as a hyphal-inducing signal, which impinges on the Efg1 signaling pathway, suggesting that the BHT inhibitors were affecting this signaling pathway. This hypothesis was supported by hyphal-specific gene expression assays using a GFP reporter expressed under the control of the *HWP1* promoter (*HWP1pr*-GFP; [37]). *HWP1* expression is regulated primarily through the Efg1 signaling pathway [38], and all the BHT inhibitors, except GW 5074, could inhibit *HWP1pr*-GFP expression [35], [36].

The effects of a subset of the BHT inhibitors (Fig. 1B) in different hyphal-inducing media, as well as in chemical epistasis experiments with constitutively active mutants, were determined in order to address the specific signaling pathways affected by these BHT inhibitors. The data suggest that the BHT inhibitors primarily affect the Efg1 pathway but some have effects on other, or multiple, pathways. Of particular note is clozapine, a FDA-approved anti-psychotic drug that inhibits dopamine receptors in the brain [39]. Dopamine receptors belong to the family of G-protein-coupled receptors that regulate cAMP levels in mammalian cells [40]. Various clozapine metabolites and functional derivatives are also able to inhibit the BHT, with the likely site of action being the G-protein-coupled receptor Gpr1, which mediates a nutrient response through the cAMP-dependent Efg1 signaling pathway.

## Results

### Effects of BHT inhibitors in hyphal-inducing media

**Figure 2:**
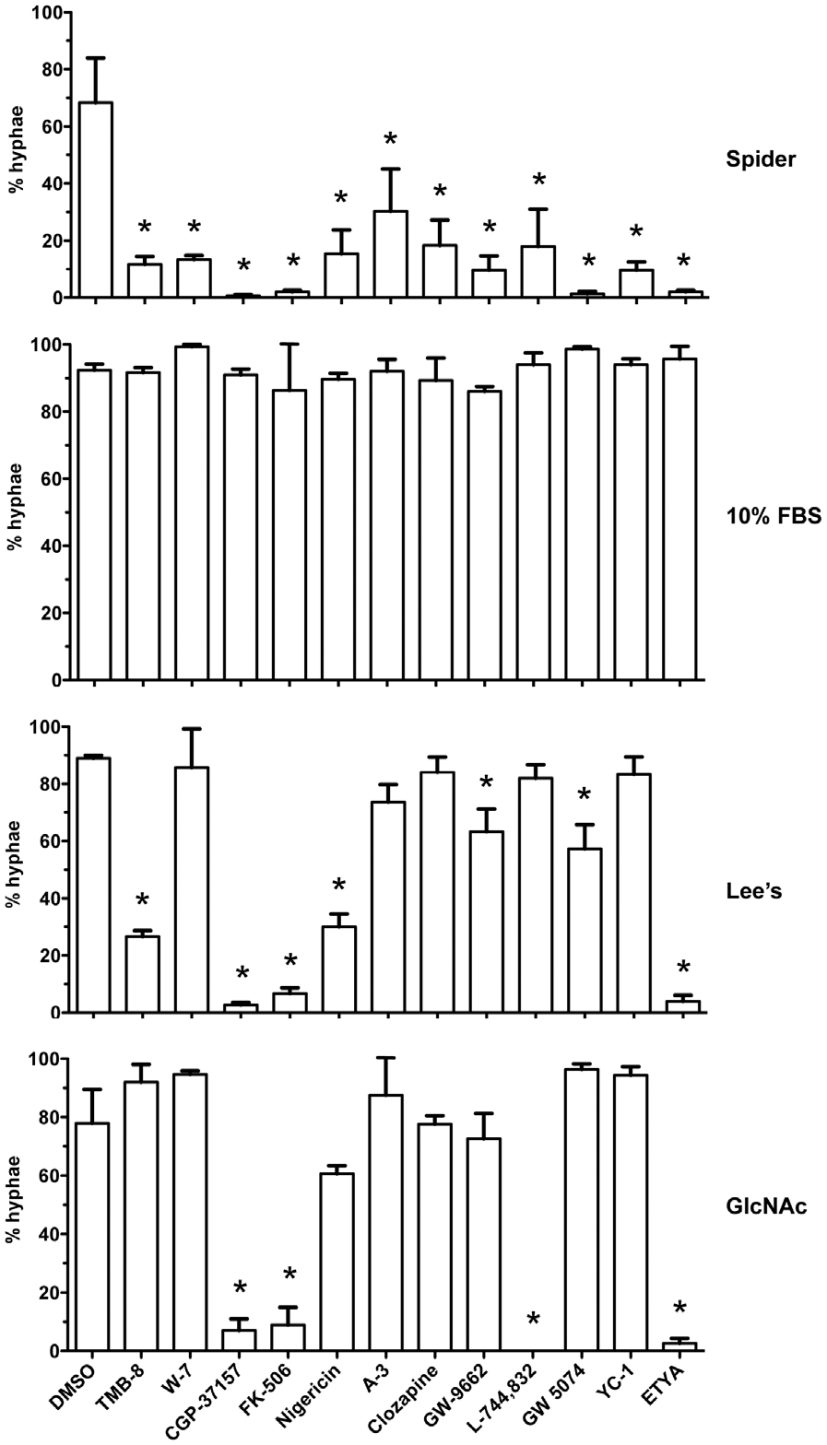
BHT inhibition in different hyphal-inducing media. Wild-type strain SC5314 was grown in the four different hyphal-inducing media for 6 h at 37°C. At least 100 cells were counted for each sample in duplicate and all assays were repeated at least twice. Standard deviations are shown for each sample. Asterisks indicates a *P* value of <0.05 compared to the DMSO control.

The effects of 12 BHT inhibitors (Fig. 1B) were tested under different hyphal-inducing conditions. The hyphal-inducing media used were (*i*) carbon-limiting Spider media (the original screening media); (*ii*) complex YPD media+10% serum; (*iii*) minimal amino acids Lee's media; and (*iv*) minimal media containing GlcNAc as the sole carbon source. All of the inhibitors were effective in Spider media (as previously reported; [36]), but none were effective in YPD+10% serum (Fig. 2), which may be the result of lipid-binding serum constituents binding and inactivating the BHT inhibitors. CGP-37157, FK-506, and ETYA were equally effective in Spider, Lee's, and GlcNAc media, suggesting that these molecules are either not affecting a specific nutrient-signaling pathway or inhibiting a common component of multiple signaling pathways. TMB-8, nigericin, GW-9662, and GW 5074 were the only other molecules that were effective in Lee's media; these molecules may inhibit an upstream component of the Cph2/Tec1 pathway. L-744,832 was the only other BHT inhibitor capable of blocking GlcNAc-induced BHT, suggesting that this molecule may affect a component of this ill-defined pathway. These data suggest that although all the inhibitors are affecting the Efg1 pathway, which is not surprising given that the original screen was in Spider media, some of the inhibitors have effects on other signaling pathways as well.

### Chemical epistasis studies

Chemical epistasis studies were used to determine if a BHT inhibitor acted at a particular step in a signaling pathway. This method involved combining an inhibitor with a constitutively active mutant within a pathway. The phenotype of the inhibitor (budded growth) is opposite of the phenotype of a constitutively active mutant (hyphal growth). When combined, if the new observed phenotype is that of the constitutively active mutant, then the mutant is epistatic to the inhibitor and, hence, the inhibitor likely acts upstream of the mutant in the signaling pathway. However, if the new observed phenotype is that of the inhibitor, then the inhibitor is epistatic to the constitutively active mutant, suggesting that the inhibitor acts downstream of the mutant or in another pathway.

**Figure 3:**
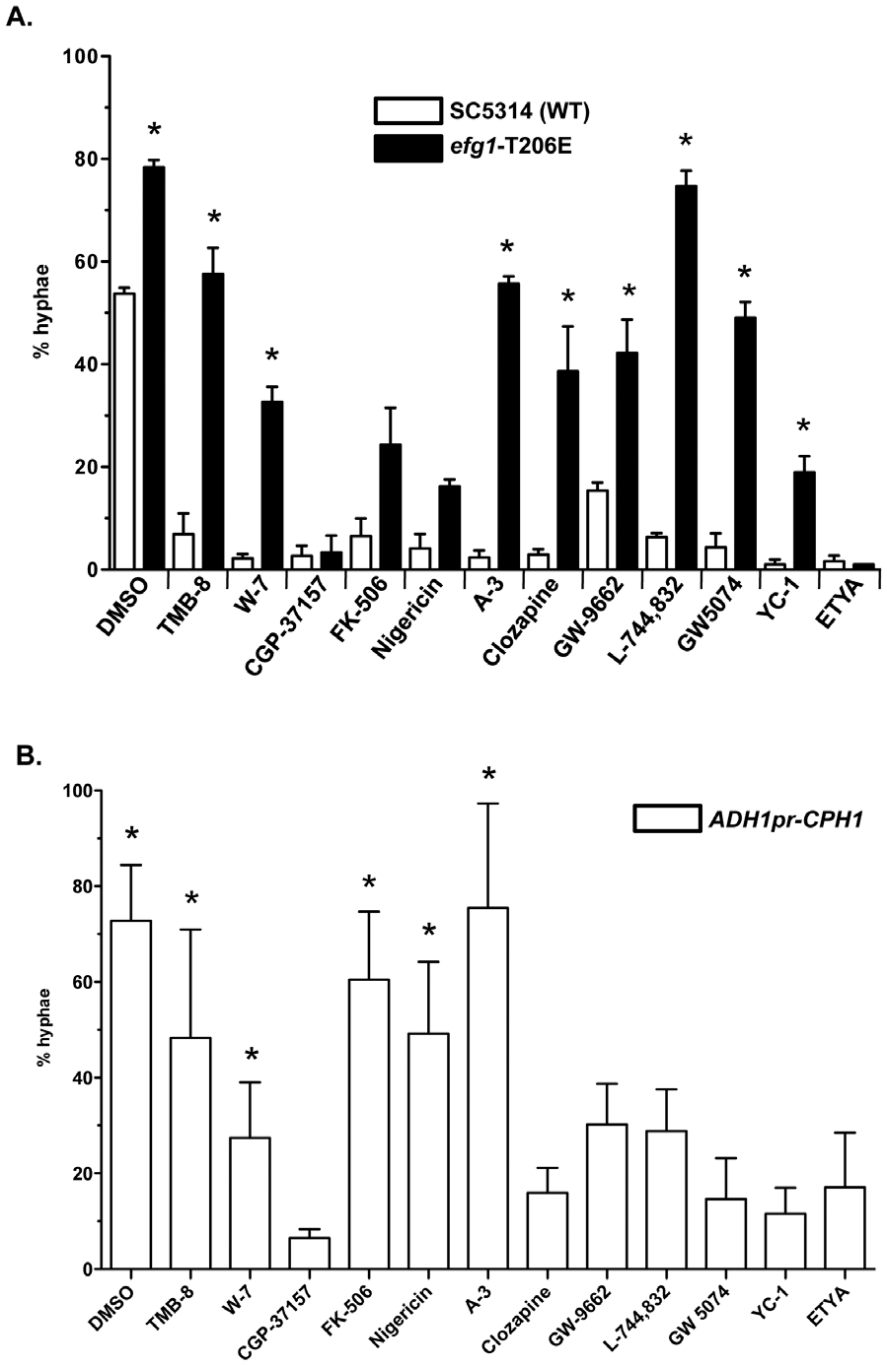
Chemical epistasis with *efg1*-T206E mutant and *ADH1pr-CPH1* overexpression. **A,** Wild-type strain SC5314 (white bars) and *efg1*-T206E mutant strain AV55 (black bars) were grown in Spider media with the indicated BHT inhibitors (100 µM final concentration) for 6 h at 37°C prior to quantification of morphological phenotype as in Figure 2. Asterisks indicates a *P* value of <0.05 compared to the corresponding wild-type SC5314 plus BHT inhibitor control. **B,** *ADH1pr-CPH1* strain CDH72-1 was assayed in the presence of the indicated BHT inhibitors as in A. Asterisks indicates a *P* value of <0.05 compared to the wild-type SC5314 plus BHT inhibitor control.

Since all of the BHT inhibitors are predicted to inhibit the Efg1 pathway, the first chemical epistasis experiments were performed with the constitutively active phosphomimic *efg1*-T206E mutant. The Efg1 transcription factor is regulated by cAMP-dependent protein kinase A (PKA) [41], which is encoded by two catalytic subunits in *C. albicans*, Tpk1 and Tpk2 [16], [42]. Efg1 contains a PKA consensus phosphorylation site at Thr206 and the phosphomimic *efg1*-T206E mutation was constitutively active, displaying a hyperfilamentation phenotype [41], suggesting that phosphorylation of Efg1 at Thr206 was important for induction of the BHT. This hyperfilamentation phenotype was observed versus the SC5314 wild-type strain (Fig. 3A, DMSO control, 79% vs. 54%; *P*<0.05). Therefore, observation of the *efg1*-T206E constitutive hyperfilamentation mutant phenotype in the presence of a BHT inhibitor would suggest that the inhibitor acts upstream of Efg1 in the pathway.

CGP-37157, ETYA, nigericin, and FK-506 were epistatic to the constitutively active *efg1*-T206E mutant (*i.e.*, displayed a budded phenotype) (Fig. 3A), which suggested that these BHT inhibitors either act in other signaling pathways, as was predicted from the hyphal-inducing media studies (see above), or downstream of Efg1. The *efg1*-T206E mutant was epistatic to the remainder of the BHT inhibitors tested (Fig. 3A). These data suggested that these molecules may act upstream of the Efg1 transcription factor.

If these BHT inhibitors were only affecting the Efg1 signaling pathway, then overexpression of the Cph1 transcription factor in the Cek1 MAPK pathway (Fig. 1B) would be predicted to have little or no effect on the BHT inhibitor activity. Overexpression of the Cph1 transcription factor under the control of the constitutive *ADH1* promoter (*ADH1pr-CPH1*; [13]) had no statistically significant effect on the inhibitory activity of CPG-37157, clozapine, GW-9662, L-744,832, GW 5074, YC-1, and ETYA (Fig. 3B), reinforcing the notion that CPG-37157 and ETYA inhibit other signaling pathways (see above) and that clozapine, GW-9662, L-744,832, GW 5074, and YC-1 act primarily through the Efg1 pathway. Overexpression of Cph1 suppressed the effects of TMB-8, W-7, FK-506, nigericin, and A-3, suggesting that these BHT inhibitors may be affecting components of the Cph1 pathway.

**Figure 4:**
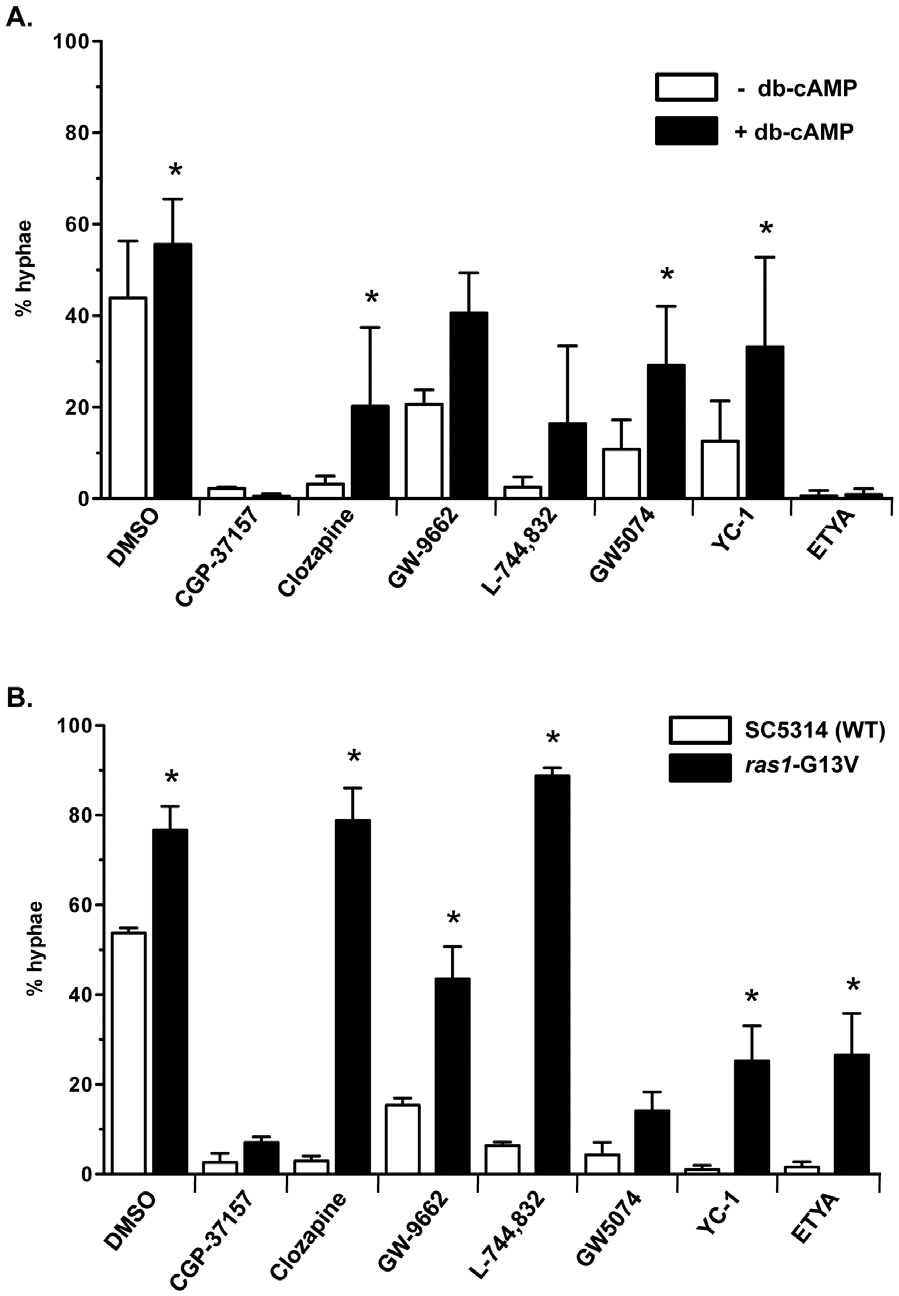
Chemical epistasis with cAMP and *ras1*-G13V mutant. **A,** Wild-type strain SC5314 was incubated in the absence (white bars) and presence (black bars) of 10 mM *N* ^6^,2′-*O*-dibutyryl-cAMP (db-cAMP) for 6 h at 37°C. Quantification of morphological phenotypes was as in Figure 2. Asterisks indicates a *P* value of <0.05 compared to the corresponding –db-cAMP control. **B,** The *ras1*-G13V constitutively active mutant strain DH409 was incubated in Spider media with the indicated BHT inhibitors for 6 h at 37°C as in Figure 3. Asterisks indicates a *P* value of <0.05 compared to the corresponding wild-type SC5314 plus BHT inhibitor control.

Given that clozapine, GW-9662, L-744,832, GW 5074, and YC-1 may act upstream of Efg1, possibly at the level of the PKA subunits or Cyr1/adenylyl cyclase, similar epistasis experiments were performed using added db-cAMP. Addition of 10 mM *N* ^6^,2′-*O*-dibutyryl-cAMP (db-cAMP), a non-metabolizable derivative of cAMP, resulted in increased filamentation (DMSO control, Fig. 4A), and was epistatic to the addition of clozapine, GW 5074, and YC-1, but had no effect on CGP-37157 and ETYA inhibitory activity (Fig. 4A). There was an effect of added db-cAMP on GW-9662 and L-744,832 treated cells, but it was not statistically significant. These data suggest that clozapine, GW 5074, YC-1 and possibly GW-9662 and L-744,832 act upstream of adenylyl cyclase in the pathway.

The Ras1 GTPase controls the Efg1 pathway independently of the Gpr1 receptor and it also regulates the Cek1 MAPK pathway leading to activation of the Cph1 transcription factor (Fig. 1A). The constitutively active *ras1*-G13V mutant [11] displayed increased filamentation (DMSO control, Fig. 4B) that was epistatic to addition of clozapine, GW-9662, L-744,832, YC-1 and surprisingly ETYA, but not CGP-37157 or GW5074 (Fig. 4B). These data suggest that clozapine, GW-9662, L-744,832, and YC-1 may act upstream of Ras1. ETYA was epistatic to the *efg1*-T206E mutant and added db-cAMP but not to the *ras1*-G13V mutant, suggesting that ETYA is acting upstream of Ras1 but not through the Efg1 pathway.

**Figure 5:**
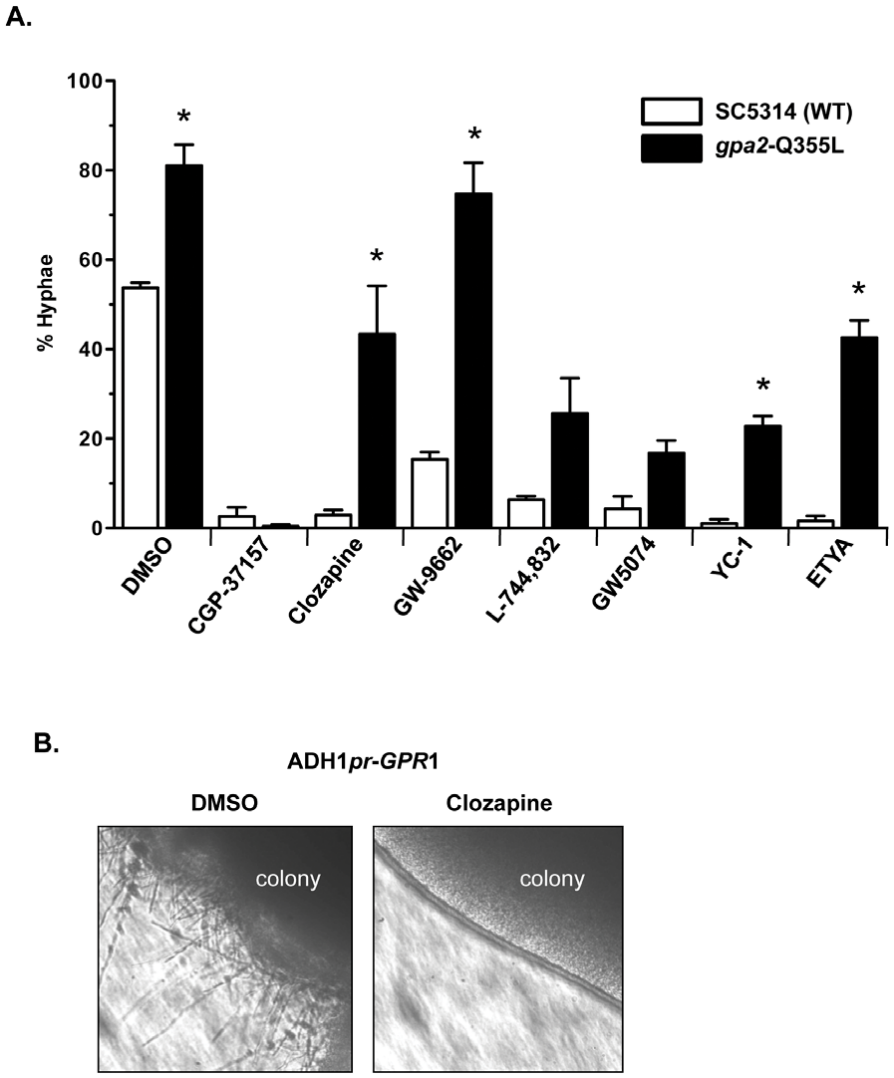
Chemical epistasis with *gpa2*-Q355L mutant and *ADH1pr-GPR1* overexpression. **A,** Wild-type strain SC5314 (white bars) and *gpa2*-Q355L mutant strain HTC7 (black bars) were grown in Spider media with the indicated BHT inhibitors (100 µM final concentration) for 6 h at 37°C prior to quantification of morphological phenotype as in Figure 2. Asterisks indicates a *P* value of <0.05 compared to corresponding wild-type SC5314 plus BHT inhibitor control. **B,** The *ADH1pr-GPR1* strain YTC014 was spotted onto Spider solid media containing either DMSO or clozapine (∼100 µM final concentration) and incubated for 7 days at 37°C.

The G-protein coupled receptor Gpr1 interacts with the G-alpha subunit Gpa2 to mediate glucose-limiting hyphal-inducing signals in the Efg1 signaling pathway. Overexpression of Gpr1 under the control of the constitutively active *ADH1* promoter leads to hyperfilamentation on solid Spider media, but not liquid media, whereas expression of the constitutively active *gpa2*-Q355L mutant results in increased filamentation in liquid Spider media [19]. If the BHT inhibitors act through inhibition of Gpr1, then the combination of the BHT inhibitor with ADH1*pr*-*GPR1* should show a lack of hyperfilamentation on solid Spider media, and combination with the *gpa2*-Q355L mutant should show a hyperfilamentation phenotype in liquid Spider media. The *gpa2*-Q355L mutant was epistatic to addition of clozapine, GW-9662, and YC-1, but not L-744,832 (Fig. 5A), suggesting that clozapine, GW-9662, and YC-1 may act upstream of Gpa2, presumably at the level of the Gpr1 G-protein coupled receptor and L-744,832 acts at the level between Ras1 and Gpa2. The *gpa2*-Q355L mutant was also epistatic to the addition of ETYA as was the *ras1*-G13V mutant, suggesting that EYTA may have an effect upstream of Ras1 and Gpa2 that is independent of the Efg1 pathway.

Constitutively active *gpr1* mutants do not exist currently, but overexpression of *GPR1* under the control of the ADH1 promoter (*ADH1pr-GPR1*) does leads to a hyperfilamentation phenotype on solid Spider media [19]. Interestingly, addition of clozapine can reverse this hyperfilamentation phenotype on solid media (Fig. 5B), suggesting that clozapine can inhibit the function of the Gpr1 G-protein coupled receptor.

### Activity of clozapine derivatives on the BHT

**Figure 6:**
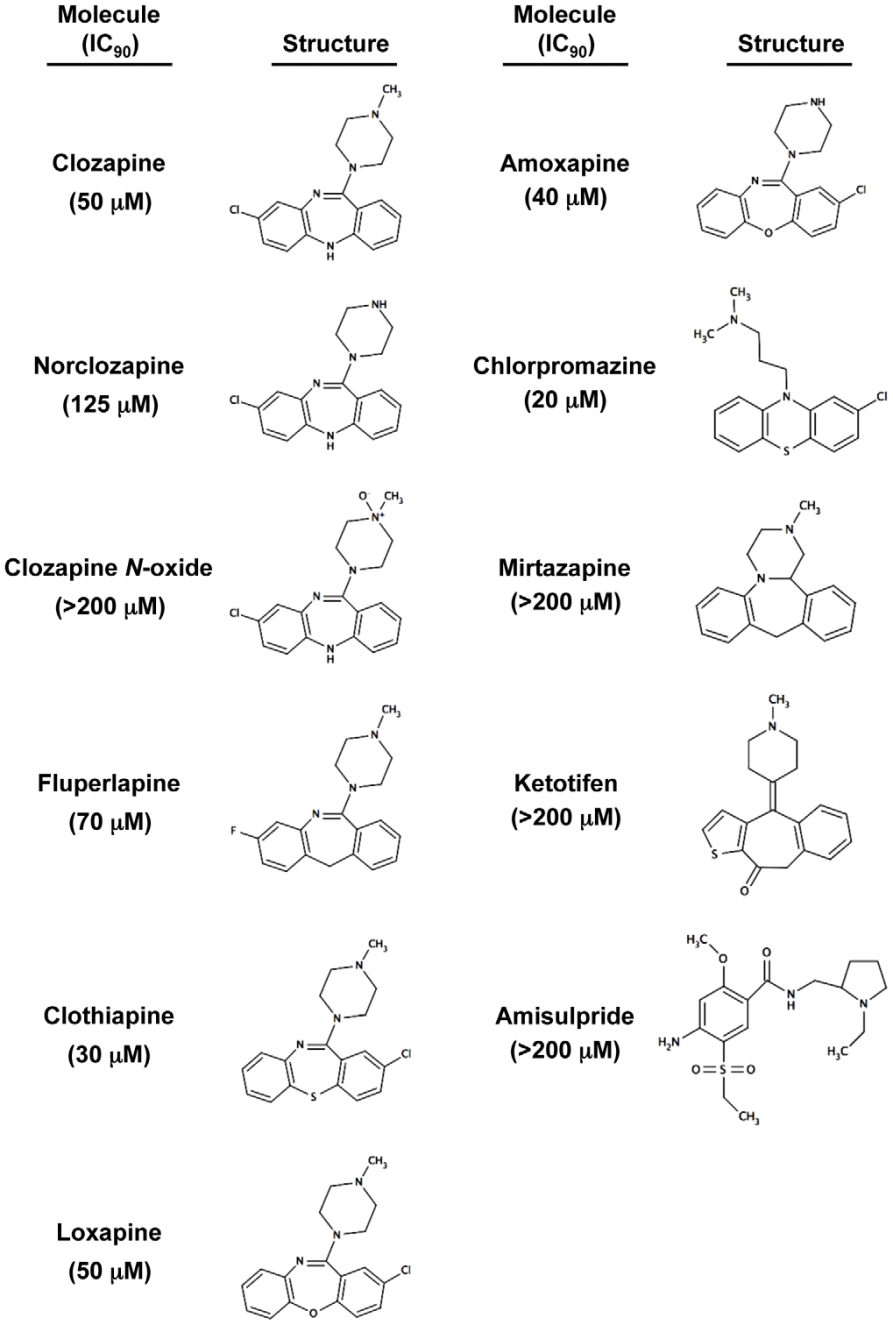
Clozapine and its bioactive structural derivatives inhibit the BHT. The indicated molecules were tested in the BHT morphological assay in Spider media for 6 h at 37°C. Below each molecule name is their IC_90_ value (*i.e.*, lowest concentration of molecule used at which >90% budded cells were observed), which was determined using serial dilutions of the individual molecules. Those molecules with IC_90_ values over 200 µM were not inhibitory in the BHT assay.

Clozapine inhibited *HWP1pr*-GFP expression [36] and blocked the hyperfilamentation associated with *GPR1* overexpression (Fig. 5B), suggesting that clozapine is inhibiting the Efg1 signaling pathway at the level of the Gpr1 receptor. Clozapine is an FDA-approved anti-psychotic drug that inhibits G-protein-coupled dopamine receptors. Commercially available structural derivatives of clozapine (Fig. 6) were analyzed in order to identify potential structural determinants necessary for clozapine activity. Norclozapine was able to inhibit the BHT but with a higher IC_90_ of 125 µM, whereas clozapine *N*-oxide was ineffective at concentrations up to 200 µM. Fluperlapine, clothiapine, loxapine, amoxapine, and chlorpromazine, which have either anti-psychotic or anti-depressant clinical utility, also inhibited the BHT, with the most effective being chlorpromazine (IC_90_ 20 µM). Fluperlapine also displayed the same chemical epistasis phenotypes with the *efg1*-T206E, db-cAMP, *ras1*-G13V, and *gpa2*-Q355L mutants as clozapine (data not shown), suggesting that fluperlapine is acting by a similar mechanism. It should be noted, however, that BHT inhibition is not associated with all anti-psychotic or anti-depressants that are receptor antagonists, as mirtazapine (serotonin and histamine receptors), ketotifen (histamine receptors) and amisulpride (dopamine D2 and D3 receptors) did not inhibit the BHT at concentrations up to 200 µM.

## Discussion

Taken together, the BHT inhibition and epistasis data (Table 1) allow for several conclusions. CGP-37157 does not inhibit a component of either the Cph1 or Efg1 pathway. CGP-37157 is known to affect calcium homeostasis through regulating intracellular calcium levels [43], a global effect that may not occur through a single signaling pathway. TMB-8, FK-506, W-7, and nigericin are also known to affect calcium homeostasis in the cell, either through affecting calcium/calmodulin-dependent protein kinase [W-7; [44], the calcineurin phosphatase [FK-506; [45], or intracellular calcium levels [TMB-8; [46] and nigericin; [47]. Of these four BHT inhibitors, only TMB-8 can block the BHT in Lee's media, and only FK-506 can block in GlcNAc media, suggesting that these four inhibitors are blocking different pathways responding to different growth signals. All four inhibitors are suppressed by overexpression of the Cph1 transcription factor and by the *efg1*-T206E mutant. Taken together, these data suggest that TMB-8, W-7, FK-506, and nigericin affect multiple signaling pathways, including the Efg1 and Cph1 pathways, presumably by affecting calcium homeostasis.

**Table 1:** Summary of BHT inhibition and chemical epistasis data.

Molecule	Spider	10% serum	Lee's	GlcNAc	*efg1*-T206E	*ADH1pr-CPH1*	db-cAMP	*ras1*-G13V	*gpa2*-Q355L	Predicted Pathway
TMB-8	+	−	+	−	+	+	NT	NT	NT	multiple
W-7	+	−	−	−	+	+	NT	NT	NT	multiple
CGP-37157	+	−	+	+	−	−	−	−	−	multiple
FK-506	+	−	+	+	−	+	NT	NT	NT	multiple
Nigericin	+	−	+	−	−	+	NT	NT	NT	multiple
A-3	+	−	−	−	+	+	NT	NT	NT	Efg1, Cph1
Clozapine	+	−	−	−	+	−	+	+	+	Efg1
GW-9662	+	−	+	−	+	−	−	+	+	Efg1
L-744,832	+	−	−	+	+	−	−	+	−	Efg1
GW 5074	+	−	+	−	+	−	+	−	−	Efg1
YC-1	+	−	−	−	+	−	+	+	+	Efg1
ETYA	+	−	+	+	−	−	−	+	+	multiple

In the Spider, 10% serum, Lee's, and GlcNAc columns, “+” refers to statistically significant BHT inhibitory activity (*P* value of <0.05 compared to the DMSO control; see Figure 2), whereas “−” refers to no statistically significant BHT inhibitory activity (*P* value of >0.05 compared to the DMSO control). In the *efg1*-T206E, *ADH1pr-CPH1*, db-cAMP, *ras1*-G13V, and *gpa2*-Q355L columns, “+” refers to the constitutively active mutant or db-cAMP being epistatic to the added BHT inhibitor, whereas “−” refers to the added BHT inhibitor being epistatic to the constitutively active mutant or db-cAMP. NT, not tested.

ETYA inhibited in Spider, Lee's and GlcNAc media, suggesting that it can block multiple signaling pathways like CPG-37157. However, while ETYA was epistatic to *ADH1pr-CPH1* expression, the *efg1*-T206E mutant, and added db-cAMP, it was not epistatic to the *ras1*-G13V or *gpa2*-Q355L constitutively active mutants. Taken together, these data suggest that ETYA is acting upstream of Ras1 and Gpa2 but not through the Efg1 or Cph1 signaling pathways. ETYA is a member of the oxylipin family of oxidized fatty acids and is the alkyne homolog of arachidonic acid. It is a known inhibitor of mammalian lipooxygenases (LOXs) and cyclooxygenases (COXs) [48], [49], which convert arachidonic acid to eicosanoids, such as prostaglandins and other bioactive lipids. ETYA has also been shown to be a potent activator of the peroxisome proliferator-activated receptor alpha (PPAR-alpha), a nuclear receptor transcription factor that activates the expression of multiple genes involved in the cellular uptake, activation, and beta-oxidation of fatty acids [50]–[52]. In addition, ETYA has been shown to possess anti-oxidant capabilities in mammalian and plant cells [53], [54], and is structurally similar to farnesol and farnesoic acid, two known quorum-sensing molecules in *C. albicans* [55], [56]. Therefore, ETYA may be inhibiting the BHT through a variety of diverse regulatory mechanisms. *C. albicans* cells produce a variety of oxylipins, eicosanoids, and other bioactive lipids that regulate cell growth, morphogenesis, biofilm formation, and virulence [57]–[60], but the mechanisms by which this regulation occur are unknown. The absence of recognizable LOXs and COXs in the annotated *C. albicans* genome compounds the uncertainty surrounding the mechanism of action of ETYA in *C. albicans*.

A-3 was only effective in Spider media but was suppressed by overexpression of the Cph1 transcription factor and by the *efg1*-T206E mutant, suggesting that A-3 may be inhibiting a component of both pathways. A-3 has a broad inhibitory spectrum against protein kinases but seems to have better efficacy against protein kinase A (PKA) [61], which was particularly encouraging as the role of PKA in the Efg1 pathway is well established [16]. A-3 may have inhibitory activity against one of the MAP kinases in the Cph1 pathway as well, which will be tested in the future.

L-744,832 was inhibitory in both Spider and GlcNAc and was suppressed by the *efg1*-T206E mutant but not by overexpression of the Cph1 transcription factor, suggesting that it inhibits a component of the Efg1 pathway. It was also suppressed by the *ras1*-G13V mutant but not by the *gpa2*-Q355L mutant, suggesting that it is acting at the level of Ras1. This is consistent with the fact that L-744,832 is a known farnesyltransferase inhibitor [62] that can block Ras function as well as other farnesylated proteins in the cell. Interestingly, L-744,832 may be inhibiting a farnesylated protein(s) that functions within the ill-defined GlcNAc pathway.

Clozapine and YC-1 were only inhibitory in Spider media whereas GW-9662 and GW 5074 also blocked in Lee's media. All four BHT inhibitors were suppressed by the *efg1*-T206E mutant but not by overexpression of the Cph1 transcription factor. These data suggest that clozapine, GW-9662, GW 5074, and YC-1 inhibit components within the Efg1 signaling pathway. The target of the guanylyl cyclase activator YC-1 in the Efg1 pathway is unclear as there is no recognizable guanylyl cyclase encoded in the *C. albicans* genome. GW 5074 effects were reversed by added db-cAMP but not by the *ras1*-G13V or *gpa2*-Q355L constitutive mutants, suggesting that GW 5074 is acting at a level between Ras1 and adenylate cyclase. GW5074 inhibits the mammalian cRaf-1 kinase [63], which functions downstream of the Ras GTPase. Although a Raf-1-like kinase as not been identified in the Efg1 pathway in *C. albicans* to date, these data would suggest that a similar protein functions downstream of Ras1 in the Efg1 pathway but not the Cph1 pathway.

Both clozapine and GW-9662 are mammalian receptor antagonists. The epistasis data suggest that clozapine and GW-9662 inhibit a component of the Efg1 pathway that is upstream of the Gpa2 G-alpha subunit, possibly the Gpr1 G-protein-coupled receptor (GPCR). The clozapine inhibitory effects seem to be specific in that (*i*) structural derivatives that are active in mammalian cells are inhibitory in *C. albicans*; (*ii*) the inactive analog clozapine *N*-oxide is inactive in *C. albicans*; and (*iii*) three other receptor antagonists (mirtazapine, ketotifen, and amisulpride) are not inhibitory in *C. albicans*. Although clozapine can suppress the hyperfilamentation phenotype of overexpression of Gpr1, it remains to be determined if clozapine directly binds to the Gpr1 GPCR to inhibit its function, as it does with the mammalian dopamine receptors.

From a structural perspective, the data from the clozapine analogs suggest several things. The *N*-methyl group of clozapine is important but not essential for it inhibitory function because norclozapine had reduced efficacy but could still inhibit the BHT and because amoxapine (IC_90_ 40 µM) had comparable efficacy to its methylated analog loxapine (IC_90_ 50 µM). Also, replacement of one of the nitrogens in the benzodiazepine ring system with sulfur (clothapine and chlorpromazine) increases the efficacy about 2-fold, which may be reflective of either increased binding avidity or molecule stability.

Although all 16 BHT inhibitors were isolated in screens using the carbon-limiting hyphal-inducing signal of Spider media, implying they would be inhibiting a component of the Efg1 pathway, it is clear that some of the inhibitors have effects on other signaling pathways as well. These effects may be indicative of cross-talk between pathways or of common components that function in multiple pathways. Identifying the *C. albicans* cellular targets of these inhibitors should clarify these points in the future.

## Materials and Methods

### Growth Media and Strains

Protocols for the growth and maintenance of *C. albicans* strains were described previously [35], [36]. The hyphal-inducing media used in these studies were (*i*) carbon-limiting Spider medium [[7]; 1% nutrient broth, 1% mannitol, 0.2% K_2_HPO_4_], (*ii*) YPD (1% yeast extract, 2% peptone, and 2% dextrose)+10% (vol/vol) fetal calf serum, (*iii*) minimal amino acids Lee's medium [6]; contains the amino acids alanine, leucine, lysine, methionine, phenylalanine, proline, and threonine+1% dextrose], and (*iv*) GlcNAc (NPK) media [[24]; 0.5% GlcNAc, 0.5% peptone, 0.3% KH_2_PO_4_].

The *Candida albicans* strains used in this study are listed in Table 2. Strain AV55, which contains the *efg1*-T206E allele under the control of the *PCK1* promoter [41], was constructed by transforming strain CAI4 (*ura3*::λ*imm434*/*ura3*::λ*imm434*; [64]) with linearized plasmid pDB2 [41], directing integration of *PCK1pr-efg1*-T206E at the *LEU2* locus.

**Table 2:** *C. albicans strains*.

Strain	Genotype	Reference
SC5314	Wild-type clinical isolate	[66]
AV55	*ura3*::λ*imm434*/*ura3*::λ*imm434; LEU2::pCK1-efg1-T206E::URA3*	This study
DH409	*ura3*::λ*imm434*/*ura3; ras1-G13V*	[56]
HTC7	*ura3*::λ*imm434*/*ura3*::λ*imm434; TRP1/TRP1::ADH1pr-gpa2-Q355L::URA3*	[19]
YTC014	*ura3*::λ*imm434*/*ura3*::λ*imm434; TRP1/TRP1::ADH1pr-GPR1-URA3*	[19]
CDH72-1	*ura3/ura3 cph1*Δ::*hisG/cph1*Δ::*hisG; ADH1pr-CPH1*	[13]

### BHT Inhibitors

All small molecule BHT inhibitors were dissolved in DMSO to a stock concentration of 40 mM (except for nigericin, FK-506, and L-744,832, which were dissolved to a stock concentration of 20 mM) and stored at 0°C. Molecules clozapine, clozapine-*N*-oxide, norclozapine, clothiapine, fluperlapine, ketotifen, A-3, and ETYA were purchased from BIOMOL International/Enzo Life Sciences (Plymouth Meeting, PA); molecules TMB-8, GW 5074, GW-9662, FK-506, loxapine, chlorpromazine, amoxapine, YC-1, and *N* ^6^,2′-*O*-dibutyryl-cAMP (db-cAMP) were purchased from Sigma-Aldrich (St. Louis, MO); and molecules W-7, L-744,832, amisulpride, mirtazapine, and CGP-37157 were purchased from Tocris Bioscience (Ellisville, MO). Unless otherwise noted in the text, all small molecules were used at a final concentration of 100 µM in liquid BHT and chemical epistasis assays.

### BHT and chemical epistasis assays

The BHT morphology assay in different hyphal-inducing media was performed as previously described [35]. *C. albicans* SC5314 cells were grown overnight in 5-ml cultures of YPD media at 30°C to block hyphal formation. Cells were washed once with ddH_2_O and an aliquot of cells was inoculated into YNB media (0.67% yeast nitrogen base, 2% dextrose, and the required supplements of uridine, histidine, and arginine, pH 7) and grown to O.D. of 1.0, then washed once with ddH_2_O. Approximately 1×10^4^ cells were then added to each of the four hyphal-inducing media and incubated at 37°C for 6 h. After 6 h, the morphology of cells was determined by light microscopy.

Quantification of BHT inhibition was accomplished by counting the number of individual budded cells versus the number of hyphae in the population as previously described [35]. More than 100 cells were counted for each sample in duplicate and all assays were repeated at least twice. Large-budded cells (i.e., buds were comparable in size to the mother cells) were counted as two cells, while small-budded cells (i.e., buds were smaller than the mother cells) were counted as one cell. This method, which was necessitated by the aggregation of budded cells that could not be resolved by sonication, resulted in an underrepresentation of the number of budded cells in the population. Individual hyphae were counted as one cell, although the hyphae usually consisted of multiple individual hyphal cells. This method, which was necessitated by the inability to quantify individual cells within hyphae, also resulted in an underrepresentation of the number of hyphal cells in the population. The IC_90_ value for selected BHT inhibitory molecule (*i.e.*, lowest concentration of molecule used at which >90% budded cells were observed) was determined using serial dilutions of the individual molecules.

The chemical epistasis assays were performed similarly to the BHT assays. For the *efg1*-T206E epistasis experiments, strain AV55 was grown overnight in SC+AA ([65]; 0.67% yeast nitrogen base plus amino acids) to de-repress the *PCK1* promoter, followed by incubation in Spider media with added BHT inhibitor (final concentration 100 µM) for 6 h at 37°C. After 6 h, the morphology of cells was determined by light microscopy. For the cAMP epistasis experiments, strain SC5314 was incubated in Spider media +/−10 mM *N* ^6^,2′-*O*-dibutyryl-cAMP (db-cAMP) for 6 h at 37°C. Epistasis experiments with the *ras1*-G13V and *gpa2*-Q355L mutants were performed in Spider media for 6 h at 37°C.

Statistical analyses of the hyphal-inducing media experiments and the *efg1*-T206E, *ras1*-G13V, and *gpa2*-Q355L epistasis experiments were accomplished using One-way Analysis of Variance (ANOVA) with a Dunnett post test. Statistical analysis of the db-cAMP epistasis experiment was performed using a Wilcoxon matched-pairs signed rank test. All tests were performed using GraphPad Prism 3 software and data were considered significant if the *P* value was <0.05. In the hyphal-inducing media experiments, the percentage of hyphae in wild-type SC5314 cells grown in the different hyphal-inducing media plus BHT inhibitors was compared to the mean of the percentage of hyphae in wild-type SC5314 cells plus DMSO. For the *efg1*-T206E, *ras1*-G13V, and *gpa2*-Q355L epistasis experiments, the percentage of hyphae in mutant cells grown in Spider media plus BHT inhibitor was compared to the mean of the percentage of hyphae in wild-type SC5314 cells grown in Spider media plus BHT inhibitor. For the db-cAMP epistasis experiments, the percentage of hyphae in wild-type SC5314 cells grown in Spider media plus db-cAMP and BHT inhibitor was compared to the mean of the percentage of hyphae in wild-type SC5314 cells grown in Spider media plus BHT inhibitor alone.

### Hyperfilamentation on solid media assay

Either clozapine (125 µl of 40 mM solution in DMSO) or DMSO alone were spread uniformly using glass beads onto Petri plates containing 25 ml of solid Spider media. The plates were then incubated at 30°C for 3 h and at room temperature for 2 h to ensure absorption. Strain YTC014, which has the *GPR1* gene expressed under the control of the constitutive *ADH1* promoter (*ADH1pr-GPR1*), was grown overnight in YEPD, transferred to YNB as above, and then serially diluted onto the clozapine-infused plates and DMSO-infused plates. The inoculated plates were wrapped in parafilm and incubated for 7 days at 37°C. The presence of hyphal growth at the colony edges was determined using an inverted microscope.

### Microscopy

For the BHT and chemical epistasis assays, sample slides were viewed on a Nikon Eclipse E400 light microscope with a 10× objective and viewed through XF100 filter. Images were obtained by using a Spot RT Monochrome camera and viewed with Spot Advanced software (Diagnostic Instruments, Inc., Sterling Heights, MI).
